# A Space for Collaborative Creativity. How Collective Improvising Shapes ‘a Sense of Belonging’

**DOI:** 10.3389/fpsyg.2021.648770

**Published:** 2021-03-31

**Authors:** Filip Verneert, Luc Nijs, Thomas De Baets

**Affiliations:** ^1^Associated Faculty of the Arts, KU Leuven, Leuven, Belgium; ^2^Department of Music, LUCA School of Arts, Campus Lemmens, Leuven, Belgium; ^3^IPEM, Department of Musicology, Ghent University, Ghent, Belgium; ^4^CORPoREAL, Royal Conservatoire of Antwerp, Antwerp, Belgium

**Keywords:** eudaimonia, free improvisation, community music, collaborative creativity, PERMA

## Abstract

In this contribution, we draw on findings from a non-formal, community music project to elaborate on the relationship between the concept of *eudaimonia*, as defined by Seligman, the interactive dimensions of collective free improvisation, and the concept of collaborative creativity. The project revolves around The Ostend Street Orkestra (TOSO), a music ensemble within which homeless adults and individuals with a psychiatric or alcohol/drug related background engage in collective musical improvisation. Between 2017 and 2019 data was collected through open interviews and video recordings of rehearsals and performances. Participant data was analyzed through inductive analysis based on the principles of grounded theory. One interesting finding was the discrepancy in the participant interviews between social relationships indicative of a negative affect about social group interaction versus strong feelings of group coherence and belonging. Video recordings of performances and rehearsals showed clear enjoyment and pleasure while playing music. Alongside verbal reflection through one-on-one interviews video recordings and analysis of moment-to moment observations should be used, in order to capture the complexity of community music projects with homeless people. The initial open coding was aligned with the five elements of the PERMA model. Overall, we observed more focus on Relationship (sense of belonging), Engagement (flow in rehearsals and performances) and Meaning (belonging to something greater than yourself) and less on Positive Emotion and Accomplishment (goal setting).

## Introduction

According to Seligman (2011), well-being is related to *eudaimonia*, whereby flourishing emerges from the combination of Positive Emotion, Engagement, Relationships, Meaning, and Accomplishment. All of these components merge in joint music making, thus increasing the potential positive impact of focusing on well-being (Hargreaves and Lamont, 2017; Lamont et al., 2018). In recent years, community music initiatives have emerged as tools to positively impact on inclusion and participation through encouraging frameworks for interaction, influencing identity and creating opportunities for active and meaningful musical engagement (MacDonald et al., 2012). Higgins and Willingham (2017, p. 22) state that “the ascendency of community music in the academy is a result of a number of factors, including the widening of perspectives to address broader societal issues, cultural diversity and sustainability along with the role of music as an activist force and contributor to the health and well-being of its participants.”

Recent research indeed shows the importance of community music programs for establishing a *sense of belonging* and *well-being* for participants from different social, cultural and psychological backgrounds (MacDonald et al., 2012; Lee et al., 2017; Schiavio et al., 2019; Vougioukalou et al., 2019; Welch et al., 2020). This research shows how collaborative music making and improvisation can create a community and foster personal growth, social inclusion and self-realization. A number of recent studies looked at the benefits of community music programs from both the viewpoint of their *leaders* or *participant*s (Hickey, 2015; Schiavio et al., 2019; Vougioukalou et al., 2019). Their results display many positive outcomes that can easily be linked to the concept of *eudaimonia* or be viewed as beacons that mark the road to a flourishing life, such as self-expression, sense of togetherness (Schiavio et al., 2019), positive emotion and ownership (MacDonald et al., 2012), confidence building and trust (Vougioukalou et al., 2019) as well as peer friendship and empowerment (Lee et al., 2017).

In this paper, we want to contribute to these studies by looking at the participants of a community music program, The Ostend Street Orkestra (TOSO) that revolves around homeless people and individuals with a psychiatric or alcohol/drug related background. We were interested in finding out how participation in TOSO, with its specific approach may contribute to a flourishing live. Combining interviews and video observation, we explored the relationship between the concept of *eudaimonia*, as defined by Seligman in his PERMA model: Positive emotion, Engagement, (social) Relationships, Meaning and Accomplishment (Seligman, 2011), the interactive dimensions of collective free improvisations (Bailey, 1992; Nachmanovitch, 1990) and the concept of collaborative creativity (Aragon and Williams, 2011; Kenny, 2014; Bishop, 2018).

First, we describe the background of our work, adopting a PERMA (Seligman, 2011) perspective on flourishing and music making, and elaborating on free collaborative improvisation. Next, we describe TOSO, explaining its context, goal and approach. This is followed by a description of the method and the results. Finally, we discuss the results and conclude.

## Background

### Flourishing and Music Playing: A PERMA Perspective

The concept of *eudaimonia* is experiencing a renewed interest, especially in the field of music and improvisation (Dylan Smith and Silverman, 2020). Many people link playing music to joy, personal growth, challenge and contributing to something bigger than yourself. As such, playing music is a direct path to a flourishing life, and thus to *eudaimonia*. As stated by Dylan Smith and Silverman (2020, p. 4): “One of the fundamental premises of a eudaimonic orientation is that music and music learning can serve and epitomize human flourishing. The power of music to engender feelings of competence, agency, and community is extraordinary.” Quoting Elliott and Silverman (2015), the authors describe the *eudaimonic* view of music playing as an approach “in which the value of making music is largely in the fulfillment derived from the doing, the process - not in any external value that may be placed on the output” (Dylan Smith and Silverman, 2020, p. 3).

Moreover, making music together is described as one of the most empowering musical activities. Recreational music making, defined as ‘enjoyable, accessible and fulfilling group music-based activities that unite people of all ages regardless of their challenges, backgrounds, ethnicity, culture, ability or prior experience,’ has been acknowledged as having numerous individual and group benefits (Bittman et al., 2004). It is found to stimulate actions, feelings, and thoughts by group members that go beyond the music proper. Indeed, social, cultural, cognitive, affective, and physical expressions that develop naturally as groups interact through music, expand the possibilities of learning and human development (Cunha and Lorenzino, 2012; Biasutti and Mangiacotti, 2018).

Engaging in music programs is found to be beneficial for nursing home residents (Paolantonio et al., 2020) and can has a positive influence on depression symptoms and cognitive functioning in elderly participants, as found in studies from Biasutti and Mangiacotti (2018, 2021).

Not surprisingly, joint music making can be related to all the dimensions of Seligman’s PERMA model of well-being (see also Croom, 2014; Paolantonio et al., 2020). This model was the result of research in the field of positive psychology, which entails the scientific study of human flourishing and authentic happiness (Seligman and Csikszentmihalyi, 2000). In the PERMA model, Seligman (2010, 2011) combines a hedonic (a pleasant life) and eudaimonic (a good and meaningful life) perspective on well-being, defining it in terms of five elements: Positive emotions (P), Engagement (E), Relationships (R), Meaning (M), and Accomplishment (A).

*Positive emotion* is the hedonic side of happiness, defined as the presence of positive affect and the absence of negative affect (Kahneman et al., 1999). Positive emotion is related rather to enjoyment (i.e., joy, hope, empathy, gratitude) than to pleasure (i.e., immediate satisfaction of bodily needs). It is deemed a limited route because “many people are, by disposition, low in experiencing positive emotion” (website Positive Psychology Center, n.d.).

Engaging with music is a rewarding experience (Leman, 2016) and as such contributes to positive emotions (Lamont, 2012; Croom, 2014). For example, listening to music is viewed as one of the most rewarding of human experiences (Ascenso et al., 2018) and has been related to positive emotions, lower levels of reported stress and enhanced emotional regulation (Laukka, 2007; Gabrielsson, 2011; Västfjäll et al., 2012). Performing music even exceeds the effect of listening on emotion. As Nakahara et al. (2011) have shown, musical rewards or pleasurable feelings emerging from piano playing exceed those that are generated from music listening. This aligns with Huron’s assertion that music making addresses man’s primordial emotional functions to produce a wealth of compelling emotional experiences, thereby enabling musicians to engage in pleasurable emotional experiences, such as surprise, awe, chills, comfort, and even laughter (Huron, 2006). Additionally, the collaborative nature of joint music making promotes positive emotion. One of the reasons for this is the fact that joint making music involves synchronizing together while playing. Such interpersonal synchrony produces positive emotions: weakening the boundaries between the self and the group, it leads to feelings of collective joy that even enable groups to remain cohesive (Hove and Risen, 2009; Wiltermuth and Heath, 2009; Kirschner and Tomasello, 2010).

Positive emotion is intrinsically linked to high levels of engagement such as flow experience (Csikszentmihalyi, 1990). For example, Fritz and Avsec (2007) found that the experience of positive emotion during flow is related to paying less attention to the task, to having a clear idea of what actually happened, and to feeling a harmonious balance between challenges and skills. Interestingly, in their study the flow dimensions of “loss of self-consciousness” and “transformation of time” were seemingly not related to well-being in a musical context. Conversely, In the study of Habe et al. (2019), ‘transformation of time’ and ‘autotelic experience’ were higher in elite musicians compared to top athletes: the musicians experienced more flow in group than individual and satisfaction with life correlated positively with flow dimensions.

Within Seligman’s model of well-being, flow experience is clearly linked to the element of *Engagement* (Seligman, 2010, p. 236, 2011, p. 24). Flow is a mental state of focused concentration, of being fully immersed in an activity, with feelings of enjoyment and involvement (Csikszentmihalyi, 1990; Agarwal and Karahanna, 2000). It is a powerful motivational state, that is linked to, for example, self-esteem, life satisfaction, successful coping, and creativity, to name but a few.

According to Custodero (2005), playing music has the strong potential to induce a flow experience, even in infants and young children. Sinnamon et al. (2012) found that flow was frequently experienced by both amateur and advanced music students. A particularly well-suited context for experiencing flow is music improvisation (Custodero, 2002; Biasutti, 2015; Lee et al., 2017). Interestingly, Biasutti and Frezza (2009) found that musicians who are skilled in more than one instrument experience flow more easily than those who are skilled in only one instrument.

Alongside musicians’ individual experience of flow, studies have shown that making music together impacts the occurrence of flow (for an overview see, Pels et al., 2018). For example, Hytönen-Ng (2016) showed that individual’s flow experience is influenced by the presence of other members and the shared feelings inside a band. In several studies, Biasutti (2015, 2017) shows that group flow is an important element to achieve a musical product that could not be reached individually, “by responding to each other in a positive environment of cooperation that offers the students opportunities to freely express their creativity” (Biasutti, 2015 p. 9). The work of Gaggioli and colleagues on *Networked Flow* (e.g., Gaggioli et al., 2017) indicates that the key to achieving an optimal group experience is the establishment of a “collaborative zone of proximal development,” in which the being together and being able to intuitively recognize the intentions of the others (social presence) plays an important role. Gloor et al. (2013) investigated the bodily dimension of group flow. Their work suggests that getting into a synchronized swing is essential for successful collaboration. The authors focused on *honest signals* as an identifier of collaborative communication between musicians, which they consider an essential *glue* to enable the smooth operation of a creative team. The work on group flow is important, as Seligman’s approach is sometimes seen as a to individualistic vision of happiness (Arcidiacono and Di Martino, 2016).

*Relationships* encompass building of positive social relations with others, feeling socially integrated, cared about and supported by others, and a *sense of belonging* (Seligman, 2011). The relation between feeling related to others, well-being and musical participation has been found in many studies (e.g., Krause et al., 2019; Welch et al., 2020). Indeed, music making is an engaging, multisensory, and social activity (Overy and Molnar-Szakacs, 2009). Increasingly, studies are showing that making music together promotes prosocial feelings and facilitate prosocial behavior. According to Buren and colleagues (2019), three mechanisms underlie the impact of music on people’s social attitude. First, *synchronous interaction* during music playing has been found to promote collaborative cooperation (e.g., Rabinowitch and Meltzoff, 2017), helping behavior (e.g., Tunçgenç and Cohen, 2016), prosocial attitudes (e.g., Rabinowitch and Knafo-Noam, 2015), and social bonding (e.g., Launay et al., 2016). Similar effects have been reported in adults (e.g., Hove and Risen, 2009; Kokal et al., 2011). Second, playing music together requires a high degree of mutual *attention and coordination* to reach a shared goal (Kirschner and Tomasello, 2010). This shared intentionality and the accompanying motivation to engage in joint making music may provoke an intensified feeling of commitment and a sense of oneness as a group (Buren et al., 2019). Third, making music together may lead to a *heightened arousal and a better mood* (Thompson et al., 2001) and, as such promote prosocial behavior (e.g., North et al., 2004).

*Meaning* refers to a feeling of being connected to something greater than yourself. It is about answering the question of ‘why‘ we do what we do and the impact it has on others (Noble and McGrath, 2008; Seligman, 2011). Expressing oneself creatively and the pursuit of freedom through music can be seen as examples of meaning as conceived within the PERMA model (Silverman, 2020). Striving to realize one’s musical goals helps building a consistent sense of personal agency (e.g., the feeling of being able to contribute to an ensemble), structure (e.g., attending a rehearsal on a regular basis), and purpose (e.g., having a specific role within a music group) in their daily life.

In addition, making music together not only potentially reinforce those aspects, it may also provoke a shift from *I-agency to We-agency* (McNeill, 1995). According to Noble and McGrath (2008), in school contexts learners have a sense of ‘meaning’ when what they do also has an impact on others beyond themselves. In a sense, balancing and sometimes even subordinating the personal needs to the needs of the group and working toward a shared musical goal, as is often the case when making music together, may help gaining a positive perspective on, and gratitude for, your own life (Forbes and Bartlett, 2020). This is where the idea of participatory sense-making, as elaborated by De Jaegher and Di Paolo (2007), is of interest. It refers to how people make sense of each other and the world by participating in social interactions – in other words, by participating in each other’s behavior and intentions. Creating musical meaning can be an empowering process (Leman, 2016), and doing so together can positively influence the mutual experience of finding meaning or purpose in life (Croom, 2014).

Finally, *accomplishment* involves having a sense of achievement and success and goal directedness (Seligman, 2011). Musical involvement, such as engaging in rehearsals and performances, can help attain a sense of personal accomplishment (Lamont, 2012; Croom, 2014). Finding one’s role in the ensemble, experiencing growth throughout rehearsals (both individually and as a group), or giving successful concerts may contribute to this personal sense. Evidently, this also depends upon the nature of the approach taken in ensemble playing, which will be discussed in the next section, on collective free improvisation.

According to Seligman (2011), a balance between the five above elements must be achieved in order to reach a state of well-being or *eudaimonia* (Sirgy and Wu, 2009). It is assumed that people differ in how much happiness they derive from one or more of the building blocks, meaning that there are different routes to reach well-being or *eudaimonia.* Furthermore, Wong (2012) argues that other elements could be added to the PERMA model. For example, negative emotions can also lead to creativity (Wright and Pascoe, 2015) and resilience (Cho and Docherty, 2020; Sirgy, 2020), which are important factors for *eudaimonia*.

### Interactive Dimensions of Collective Free Improvisation and Collaborative Creativity

Improvisation has been defined in many different ways. Generally speaking, improvisation is defined as playing or singing something in a spontaneous and unrehearsed way, without specific preparation and often together with other musicians. As such, one common characteristic is the unpreparedness of performing music (Berliner, 1994; Azzara, 2002; MacDonald et al., 2011; Hickey, 2015). This aligns with the 4E cognition view on improvisation in which the introduction of entropy (uncontrolled musical chaos, distorting musical structure) is conceived as an important way to provoke the experience of (musical) freedom (Van der Schyff et al., 2018).

Improvisation has become one of the most integrative musical practices and is deemed beneficial in many different ways, often linking the ‘musical’ to the ‘social’ (Azzara, 2002; Sawyer, 2008; Wright and Kanellopoulos, 2010; MacDonald et al., 2011).

Recent research by Larsson and Georgii-Hemming (2019) shows that there are two main conceptualizations of improvisation, drawing on what Bailey (1992) described as ‘idiomatic’ and ‘non-idiomatic’ improvisation. This dichotomy is present in both research and (educational) practice (Hickey, 2009; Monk, 2013). On the one hand, there is structured or ‘idiomatic’ improvisation with clear goals, a focus on individual skills (Kratus, 1995; Brophy, 2001) and mostly operating within a tonal framework. On the other hand, there is free or ‘non-idiomatic’ improvisation, emphasizing the explorative process, collaboration and social interaction (Borgo, 2007; MacDonald et al., 2011; Monk, 2013). In non-idiomatic improvisation, elements such as exploration, experimentation, chaos, sound focus and collaboration initiate and shape the musical process (Nachmanovitch, 1990; Larsson and Georgii-Hemming, 2019). It is inventive, aiming at developing an attitude in which the learning outcomes are not predetermined (Hickey, 2009; Higgins and Mantie, 2013). This type of improvisation emphasizes imagination, collective interventions, the power of silence, the improper use of instruments, deep listening and dynamics (Nachmanovitch, 1990; Burnard and Dragovic, 2015). Collective free improvisation thus focuses on how participants, using musical elements and their own musical experiences, create collective meaning through verbal and non-verbal dialogue (Monk, 2013), interpersonal dynamics and communicative aspects of collaborative musical processes (Beegle, 2010; Burnard and Dragovic, 2015). In a community music context, free group improvisation is often led by one or more coaches, who as both participants and facilitators, take on an attitude of genuine interest in the value of collective free improvisation and experimentation (Higgins and Willingham, 2017; Schiavio et al., 2019). Collective free improvisation leads to better social competences, the development of meaningful relationships and creative musical thinking as well as better musical flexibility, originality and syntax (Hickey, 2009; Koutsoupidou and Hargreaves, 2009; Van der Schyff et al., 2018).

Group music making has a great potential to support *eudaimonia* by building on the social interactions (Sangiorgio and Mastnak, 2020). In the enactive view on social cognition presented by De Jaegher and Di Paolo (2007), the process of creating meaning together (participatory sense-making) starts with the interaction of individuals inside a social encounter. They take the interaction process ‘an sich’ as an autonomous process. An individual’s cognitive capacity is not enough to explain the interactional phenomena that are observed when people interact in a social encounter. Applied to joint music making, the social encounter and the freedom of improvisation leads to the creation of meaning through the interaction process. Free improvisation is an emergent feature of social encounters mediated through the bodily interactions with instruments (see Leman, 2016; Nijs, 2017; Bishop, 2018; Maes et al., 2018).

Creativity is an important element of collective improvisation and happens in the process of collaborative or participatory sense-making (De Jaegher and Di Paolo, 2007). As such, it is related to what Wright and Pascoe (2015) describe as the five paths to well-being: connect, be active, take notice, keep learning and give. it indeed happens in the moment of group encounter (Sawyer, 2006) and contributes to the creation of a ‘community’ and the emergence of collaborative creativity (Sawyer, 2008; Aragon and Williams, 2011; Burnard and Dragovic, 2015). The concept of collaborative creativity refers to the distribution of creativity across members of a group as they collaborate to solve a shared problem. When improvising and playing together as a group, new ideas can be developed through the contextual and distributed creative process (Aragon and Williams, 2011; Bishop, 2018). It gives the participants a sense of ambition and ability. As a result, a *sense of belonging* can be experienced, which is an important element of well-being.

Collective free improvisation as a pre-eminent example of collaborative creativity can potentially bring people together, engender a powerful interaction process and the forming of social skills. The musical and social interaction during free improvisation often shows emergent features e.g., expressivity, empathetic attunement to others and group flow (Monson, 1996; Sawyer, 2006; Maes et al., 2018). Kenny (2014) coins this as an example “of how musical groups can build, acquire, share and situate creativity through collaborative processes” (p. 6). This study reports feelings of flow and belonging through participatory performances (Kenny, 2014).

## The Ostend Street Orkestra (TOSO)

### Context and Goals

The Ostend Street Orkestra (TOSO) is an adult ensemble-based musical project in Ostend, Belgium. It was founded in 2014 by KleinVerhaal, a Flemish participatory arts organization, and three jazz musicians, as an artistic and positive alternative for the prevalent negative views concerning the growing group of homeless people in the city of Ostend.

Ostend is a medium-sized coastal city and municipality, located in the province of West Flanders in the Flemish Region of Belgium. Due to its success as a seaside resort and busy trade center, it combines all the assets of a large town. In recent years, the number of homeless people has soared in Ostend. Individuals gravitate to it in search of a better life, hoping to reach the United Kingdom. Few of them do manage to cross the Canal and linger in Ostend as they have no desire to return to their previous lives.

The starting point of TOSO was not founded with a view to keeping people off of the streets. The main idea was to jointly create a positive story using collective free instrumental and vocal improvisation, in which the crossover is made between different musical genres, between stage and street, between image and sound, between audience and musicians. Beyond the direct context of the activity (free improvisation) and the specificity of the group involved, the project was set up to create an egalitarian space of freedom in the immediate present (cf. Hakim Bey’s concept of a Temporary Autonomous Zone; Bey, 2003) where people could come and go as they pleased and decide what they would do and play, and how. All instruments are welcome at TOSO. Participants bring their own instrument or can borrow one from the organization. Amplifiers, drums, small percussion instruments, electric piano and a small sound system with microphones, are provided for the rehearsals and performances. The instruments used include guitar, bass, saz, drums and percussion, piano, saxophone, trumpet and vocals.

### Approach

The Ostend Street Orkestra started as a non-formal, participatory music project for the homeless, coached by three musicians. Coach 1 is a trumpet player/composer and has a background in free and structured jazz improvisation, coach 2 is a singer with a more traditionally vocal jazz training and a degree in social sciences and coach 3 is a self-taught drummer with considerable experience in free improvisation. They were chosen as coaches by the structural organization (KleinVerhaal), due to their experience and affiliation with community music projects and their ability to take up different roles, typical of community music workers such as ‘educator’ ‘facilitator, ’composer,’ ‘social worker’ (Veblen, 2007). Indeed, rather than leading the ensemble in a top-down manner, they take an egalitarian position in the project by being both participant and facilitator, and by adopting a non-apologetic attitude and displaying a genuine interest in all group members. It is the coach’s task to *enable music interactions* (Higgins and Willingham, 2017).

The three coaches work simultaneously with the Orkestra, which yields a negotiation of ideas between coach and participants, among coaches and among participants. All this is happening while playing, improvising, experimenting. Although acknowledging the importance of the structural organization (KleinVerhaal) and the musical coaches, the project started from a non-committal idea, whereby participants were not expected to have any previously acquired musical skills/experience and could come and go to the rehearsal as they pleased. Basically, the doors were always open and there was music playing. As a result, the atmosphere during the rehearsals can be somewhat chaotic and unstructured which, in fact, tends to generate the emergence of new ideas and social dynamics.

Social empowerment is enhanced by preserving dignity, energy, creativity and autonomy. To do so a space of freedom must be created in the immediate present, without membership or conditions.

The Ostend Street Orkestra has grown into a music lab with 25 musicians from motley social and musical backgrounds. This ‘music as a movement’ could have been a shortlived project but resulted in a continuous movement with labs, rehearsals, meetings, jam sessions and performances. This open mentality is reflected, among other things, in the open nature of the weekly rehearsals. People are indeed free to come and go, participate, listen and both process and the product are interchangeable. National and local government funding offered a welcome boost, but the latest developments in Belgium’s art funding policy makes prospects increasingly bleak. Project funding has indeed been cut by 60%, and government funding drastically reduced for specific initiatives in theater, music and arts in Flanders.

Gradually, TOSO grew into more than a weekly meeting and rehearsal. In 2016, film director Dany Deprez made a documentary about the TOSO project and its members’ backgrounds. That same year TOSO also made the album (CD) ‘TOSO Studio Compilation Volume I’ and entered into collaborations with Le Grand Orchestre National Lunaire from La Louvière (Belgium) and the Lucinda Ra Art Collective (a free improvisation collective). In 2018 the Orkestra opened the theater festival Theater Aan Zee with the Dadaist funeral procession ‘DANSE MASSACRE.’ In recent years the group has appeared on the stages of major cultural centers like CC De Grote Post, C-Mine, Manifiësta, Arts Centre De Vooruit as well as De Singel with a repertoire on Outsider Music. In 2019 KleinVerhaal received the ULTIMA culture prize of the Flemish government for the TOSO project.

## Method

### Objectives of the Study

The Ostend Street Orkestra is used as a case study, in an effort to explore the relationship between the concept of *eudaimonia*, as defined by Seligman in his PERMA model: Positive emotion, Engagement, social Relationships, Meaning and Accomplishment (Seligman, 2011), the interactive dimensions of collective free improvisations (Nachmanovitch, 1990; Bailey, 1992) and the concept of collaborative creativity (Aragon and Williams, 2011; Kenny, 2014; Bishop, 2018), itself used as a central notion to describe the communication of spontaneity and intuition in the TOSO performances.

The need for studies considering the relationship between musical activities and well-being for people from a broad socio-economic background, has been put forward in recent research (Lee et al., 2017; Schiavio et al., 2019; Lamont and Ranaweera, 2020). The use of the PERMA model also aligns with recent work in music education and community music (see Lee et al., 2017; Ascenso et al., 2018; Lamont and Ranaweera, 2020).

### Participants

At the time of the study, the average number of TOSO attendees was about 15 participants. However, due to changing attendance during rehearsals, only the returning members were asked to participate in this study (*n* = 12). The participants were aged between 23 and 60 (*M* = 42.5, *SD* = 13.3). Nine of them were between 40 and 60 years old and three participants between 20 and 30, with 9 male and 3 female participants. Their musical experience and education varied considerably, ranging from no music experience whatsoever to some degree of musical training (mostly autodidactic). They played the following instruments: 2 saxophones, 4 drums/percussion, 1 bass, 1 trumpet and 4 vocals. Most of the participants had a troublesome past, including homelessness, alcohol/drug abuse, psychological and psychiatric problems. To boot, society viewed them as ‘problematic,’ ‘dangerous,’ as ‘outcasts.’

### Data Collection and Analysis

Between 2017, 2019, data was collected through open interviews and video recordings of rehearsals and performances.

**FIGURE 1:**
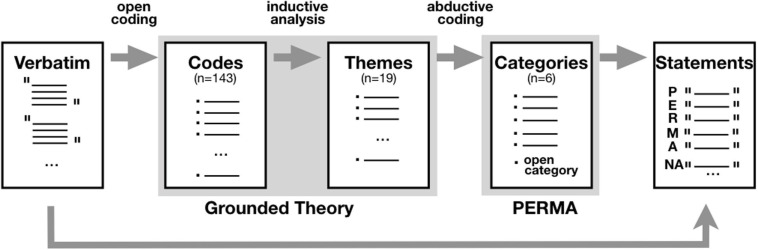
Data analysis.

The interviews (*n* = 12) were conducted before and after rehearsals. Taking into account the specific context and as such to motivate the participants to participate and speak freely, the interviews were conceived as an informal talk (recorded on a mobile phone) about the project with trainees and the primary researcher, who himself participated in some rehearsals and performances. The interviews were conducted in the third year of the program (the average duration of the interviews was 40 min with an average word count of 3100). Questions (see Appendix 1) spanned the members’ attitude toward TOSO, the social interaction and the atmosphere in the group. In an initial phase, the interviews were transcribed ad verbatim. To further analyze the interviews, we followed both a bottom-up (open coding) and top-down approach (abductive coding). First, the interview data were analyzed following a grounded theory approach. This allowed to adopt an open stance to the data, to get acquainted with the data and gain deeper insights based on the meticulous exploration of precisely transcribed data through a fluid, interactive and open-ended process (Pulla, 2014). After a close reading, the data was analyzed in ATLAS.ti (version 9.0.5) using *open* coding. Resulting codes (*n* = 143) were clustered into 19 themes using inductive analysis (Charmaz, 2006; Timonen et al., 2018). Next, following Lee et al. (2017), an *abductive* coding was performed to classify the 19 themes under the 5 categories of Seligman’s (2011) PERMA model (Positive Emotion, Engagement, Relationships, Meaning, and Accomplishment), adding an open category for themes that that did not fit the PERMA model. Finally, the statements associated with each category of the PERMA model were retrieved to bundle the main content of the quotes (see Figure 1).

To capture the complex nature of collaborative creativity and the significance of the musical and social interactions, video recordings of rehearsals and performances and a TOSO documentary were included in the analysis. Gebauer (2011) sees video recording as one of the best ways to capture musical interaction. In our study, we did not systematically record rehearsals and concerts. Apart from the documentary, the recordings were done occasionally. As such, they provide a first-hand account of ‘witnessing’ and ‘evocation’ of the practical, sensual and affective dimensions of the TOSO rehearsals and performances (e.g., Lorimer, 2010; Beresin, 2012). This is similar to an emic approach, in that it focuses on the perceptions and beliefs of TOSO members, on what is meaningful to them, and how they look at things (Kottak, 2006). Therefore, we deemed these recordings as a meaningful source of information to be triangulated with the interview data. The purpose of adding video recordings was to increase the level of knowledge and to strengthen the researcher’s standpoint from different aspects. Following Mathison (1988) and Carvalho and White (1997), we view triangulation for its potential to enrich the understanding of the participants experience with the TOSO project. Mathison asserts that researchers need to make sense of the findings and move “the focus on triangulation away from a technological solution for ensuring validity and places the responsibility with the researcher for the construction of plausible explanation about the phenomena being studied” (Mathison, 1988, p. 17).

Said recordings included 4 videos of rehearsals (total time: 2 h 25 min), an edited video recording (documentary) of TOSO (35 min) and 8 short (2 to 6 min) video recordings of concerts and recording sessions (mobile phone recordings).

An analysis and interpretation of the video sequences was performed from the viewpoint of the researcher-participant. First, the raw material was observed in detail and thematically structured in terms of musical and social interaction, adding an ‘open’ category to take into account possible unintended outcomes. As we were mainly interested in the behavior of the participants and as in these videos the verbal dimension was mostly limited to short utterances and non-verbal communication, we did not transcribe the verbal dialogues in the videos. Specific sequences were selected, on the basis that ‘something happened’ in them, a critical incident (Kelchtermans, 2010; Harrison and Lee, 2011), showing interactive musical or social behavior, discontinuities or corporeal activity, looking for what Bohnsack describes as ‘conjunctive knowledge’ or knowledge “that is embedded in the practice of action” (Bohnsack et al., 2010 p. 206). The selected sequences were then outlined using descriptive keywords (e.g., walking around while playing, eye-contact with others, smiling, talking to each other, gesturing). Those keywords were kept in a code manual, using Atlas.ti 9 (version 9.0.5) software and merged into bigger categories (e.g., bodily movement, musical interaction, social contact). Short sequences were then selected which showed exemplary features of those categories.

## Results

### Results of the Interviews

A total of 143 codes, retrieved through the open coding of the interview data, were assigned to the five categories of the PERMA model. A total of 19 themes emerged from the data. Based on the data analysis, we added a category, which was later termed ‘Negative Affect.’

The most frequently reported elements of Seligman’s PERMA model are Relationships (24%) and Meaning (20%). Quotes referring to Engagement (4%), Positive emotion (11%), and Accomplishment (11%) were described the least. Interestingly, the open category, labeled ‘negative affect,’ showed numerous (29%) reports of feeling anxious and angry. While a category of ‘negative emotion’ is included in the PERMA profiler (Butler and Kern, 2016), we found rare reports of this in studies using the PERMA model in community music projects (Lee et al., 2017; Lamont and Ranaweera, 2020).

In the following subsections, we elaborate on the themes that were categorized into the PERMA model and in the Negative Affect category. Table 1 gives an overview.

**TABLE 1:** PERMA categories + negative affect (19 themes).

Positive Emotion Quotes *n* = 16	Engagement Quotes *n* = 6	Relationship Quotes *n* = 34	Meaning Quotes *n* = 29	Accomplishment Quotes *n* = 16	Negative Affect Quotes *n* = 42
SELF ESTEEM	OUTLET	SOCIAL CONTACT IN TOSO	CONNECTION TO THE COMMUNITY	DOING YOUR OWN THING	NEGATIVE FEELINGS TOWARD OTHERS
POSITIVE FEELINGS TOWARD ACTIVITY	ABSORPTION	SOCIAL CONTACT OUTSIDE TOSO	PERCEIVED FREEDOM	SOCIAL ACHIEVEMENT	PROBLEMATIC (ALCOHOL/SOCIAL)
POSITIVE FEELINGS TOWARD GROUP		SENSE OF BELONGING FAMILY and FRIENDSHIP	MAKING SENSE OF LIFE	PERSONAL SUCCESS	NOT A REAL CONNECTION ‘I DON’T CARE’

#### Positive Emotion

Participants reported several positive emotions linked to their experiences with TOSO.

Some quotes point to an increased *self-esteem* and *self-confidence* through playing music and linking this to their personal situation. Some participants talked about “a positive, deeper experience because you have been left with a mental scar”(P2) and “breaking the loneliness”(P12), as well as the positive influence the project brought them. One of the participants commented: “It’s clearly given a bit more self-esteem to so-called social rejects or people in a precarious situation. It has boosted their self-confidence, no doubt about that”(P1).

*Positive feelings toward the activity* included the possibility of musical improvisation: “You can really improvise, and that’s my thing!”(P8) and expressing oneself freely: “Hoh, In TOSO I relax and with creativity you can express yourself and just amuse me”(P6). *Positive feelings toward the group* concerned the motivation of playing together: “Ah the joviality the commitment of the people, that they come to the rehearsals and you see they all like to play music, or sing”(P4), and the warm-heartedness inside the group: “For me, TOSO is something that stays in my heart for the rest of my life”(P6).

#### Engagement

The lowest number (4%) of quotes were found on the subject of Engagement. The two selected subthemes were *outlet* or externalizing and expressing emotions and *absorption*, which directly refers to the flow concept. An example of the latter is: “Performing establishes a mutual connection on stage but also with the audience. Whenever that happens, I am away from the world. Yes, then I am away from the world”(P4).

#### Relationships

Four themes dealt with the element of relationship, all reflecting positive relationships: *social contact in TOSO, social contact outside TOSO*, a *sense of belonging* and *family and friendship*. It’s almost as if there is a continuum, starting from more constrained examples of relationship (e.g., saying hello when rehearsals begin) to a profound feeling of friendship and family (e.g., “he’s my brother”). On the issue of social relations within the group one participant said: “Great bonds are formed here. But never forget who joins TOSO. I mean, water isn’t their drink of choice, is it?”(P5).

There were reports of social contact outside the rehearsals inspiring positive feelings toward others. One participant describes it as follows: “Before, whenever I came across people like F. in the street, I would give them a wide berth, ignore them. Now I’m interested in him as a person. I’m no longer indifferent”(P7). Some of the group members actually started playing music together, as can be deduced from the next quote: “Absolutely. I got to know someone who plays the guitar and we have since formed a duo. We also play outside the thing, and we have gigs as well.”(P9).

The *sense of belonging* subtheme refers to reports of ‘homecoming’ or the feeling of belonging to a group. Two participants said the following on the subject: “It’s not a ghetto story of ’them’ and ’us,’ no it’s ’us’ and ’them’ (laughs)”(P4). And: “It means a lot. (silence) a lot, a lot to me. Not only the friendship. It’s also a homecoming feeling for me.”(P3). The last theme (*friendship and family*) refers to building strong relationships with a high emotional value. “Me and P. have built a real, solid, fraternal friendship. How shall I put it? It’s like we’re family. P. has supported me both morally and physically. He’s become a true friend. Haven’t you, Bro’?”(P5).

#### Meaning

Three subthemes were identified inside the element of Meaning. They show how TOSO members reflect on the broader sense of significance and value of the program. Quotes show the *connection to the community* which is often connected to the act of music making. As one participant put it: “TOSO is my number one, I would drop everything to attend rehearsals, despite the fact that it’s so far. I come here by bike, rain or shine”(P8). And “Some jam sessions are disastrous, others produce this spark. You feel, ah, this is great, that’s the reason I do it”(P7).

The *perceived freedom* of playing turned out to be of paramount importance in the question *why* members take part in TOSO: “Well, yes. You can do your own thing (speaks more softly), without those endless comments, telling you do it like this or that. So, you can actually express yourself and experience that feeling of freedom”(P8). The third subtheme we identified refers to *making sense of life*, expressing feelings of loyalty, identity and value. “For me, TOSO is unique, and I am not someone who runs from group to group. I remain loyal to the group and TOSO. I have lost my heart to TOSO” (P6), and “I didn’t know I had it in me, I was always shy when I sang. I used to be introverted and unable to sing in front of an audience. That’s why, every Sunday, I travel this far (90 km) to sing” (P10).

#### Accomplishment

Quotes demonstrated the importance of social achievement. Exchanging and sharing with others in the group (e.g., about personal problems), and interacting with an audience during performances were seen as a meaningful process. One participant, talking about what he liked about performing said: “The atmosphere, performing, entertaining people and interacting with the audience”(P7). Personal success was another identified subtheme, linked to a feeling of personal accomplishment, often closely related to a feeling of positive emotion or meaningfulness. As transpired from the following statements: “TOSO in no way gives me the feeling,… that I’m part of some socio-economic project. On the contrary, people are actually being upgraded for the talents they’ve got”(P1), and “We’re all artists, beginner or not; bad or very good at it, everyone is a musician”(P5). Taking part in performances and concerts proved to be an important sign of accomplishment. Group members reported great enjoyment playing *on stage* and in concert halls, which gave them a sense of identity as musicians.

#### Negative Affect

As stated earlier, we kept an open category while coding the interviews. This revealed a number of negative emotions and expressions of hostility toward others in the group (see Table 2). We tried to capture the content in four subthemes: Negative feelings toward others in the group, referring to a problematic alcohol use and social background, not having a real social connection and an *I don’t care* attitude. Those quotes may, in a sense, seemingly contradict earlier statements, often given in the same interview.

**TABLE 2:** Negative affect.

NEGATIVE AFFECT Quotes *n* = 42			
NEGATIVE FEELINGS TOWARD OTHERS ‘If TOSO didn’t exist I would steer clear of this lot. I don’t care, there’s nothing in there, in those guys.’ P7 ‘I was told ten times today not to play. I mean, you don’t come here to be told 10 times, not now, not now, not now. Sorry hey, sorry, no, no sorry.’ P11	PROBLEMATIC (ALCOHOL – SOCIAL BACKGROUND) ‘In the beginning there was no booze, no booze during rehearsals. And now, they bring it themselves! (E. brings a backpack full) …It goes on and on. I also don’t mind the occasional drink myself, but I know when to stop.’ P7 ‘Some of them are okay but the down-and-outs, the misfits in the Orkestra are a real pain.’ P4	NO REAL CONNECTION ‘No social connections, no. This is an anti-social society anyway and people, well, like I said before. They talk to me for 10 min and then they’re off. You can’t bond with that.’ P9 ‘Well, personally, I don’t think I have a real connection with them. It’s either: Ah he’s here today, cool – or: Ah he’s not here. See what I mean? I don’t know, it’s not (thinks) exactly intimate.’ P8	‘I DON’T CARE’ ‘I come here to have fun and play, but I don’t have any friends at TOSO. When we’re together we have fun, but my real friendships are elsewhere.’ P7 ‘The minute I no longer enjoy myself I’ll stop coming. I don’t care how the project evolves, it’s no use.’ P8
‘They’re basically looking after number one, focusing on themselves. Other people don’t matter one bit.’ P3 ‘Some people in the group are not kosher and ruin the atmosphere.’ P1	‘Often there are conflicts. Mostly because some people lead marginal lives in Ostend and are homeless and, uh, here they get thrown together in a group. That’s asking for trouble.’ P3 ‘There are no real friendships because there are many losers in the group: no gumption, no backbone. They may live but it’s not like they’re alive.’ P1 ‘Alcohol is okay, but never during rehearsals. That’s bad, man. Drinking in your spare time is fine, but I myself don’t drink and drive. I mean otherwise, yeah, pfff.’ P11	‘I like to work with those people but I’m not planning on bonding with them or anything.’ P12	‘You are heard but not listened to. I basically tell myself: I’m here to do my thing, so.’ P6 ‘I’m going. I’ve had it up to here, you know what I mean?’ P11

### Results of the Video Recordings

The video analysis led to 45 coded video sequences. Five themes emerged from the data: joint corporeal activity, enjoyment and trance, peer awareness, musical interaction and social contact.

The videos of the rehearsals and concerts displayed a variety of *joint corporeal activity*, such as dancing, gestures compatible with the music, body movement, clapping, walking around while playing and imitation of behavior. It went from tiny movements in the hands, the head and the whole body, to ecstatic dancing and moving around. In many cases this had a social effect, leading to interaction between participants. For example, in one sequence, four participants started dancing in the backstage room before the gig even started, one carrying his instrument, two others holding wine bottles.

They danced in a kind of circle, passing the wine bottle, pretending to drink, making big movements with their bodies. In another sequence, during a rehearsal, some of the singers started walking around in a line, moving rhythmically to the groove. They kept doing so as the rest of the singers followed. More and more others joined in (trumpet player, drummers), walking around while playing.

A second theme concerned moments of *enjoyment*, such as smiling, cheerful expressions and excitement while rehearsing and performing. Rehearsals and performances are a non-stop event, with continuous moments of playing, creating the impression the participants were on a constant wave of sounds. Sometimes, trance-like moments were observed, i.e., when musicians did not seem to know what they played, being *in the moment*, beyond the conscious mind. For example, in one sequence, a horn player takes a microphone and starts to improvise with vocal sounds, shouting, talking and singing. He is in the middle of the group, eyes closed and holding the microphone in both hands, close to his mouth. He makes a lot of movements, even gets down to the ground. Another example shows how during a street concert, one of the musicians turns to a group of Segway users, directing them with his flute like a conductor, making them turn around in circles to the beat. In all of the selected sequences a loss of self, ecstatic behavior full of energy was observed.

A third theme concerned *peer awareness*, where musicians are constantly looking toward others, making visual eye contact while playing and sometimes initiating new ideas, and interacting.

A fourth theme was *musical interaction*. For example, a frequent observation in the selected fragments was that of someone starting a riff or a groove and the whole group slowly engaging in a musical interaction around the initial groove, which can last for a while or be changed by someone. Based on the observations of the bodily and musical reactions, this can be seen as a rewarding experience.

A final theme was *social contact*. Before and after rehearsals or concerts, social activity thrived and the social atmosphere among participants was very open. There was a lot of laughter, of telling jokes, talking to each other. When people came in, they spontaneously greeted everyone, not merely shaking hands but hugging and kissing each other. We observed eye contact and facial expressions that showed emotion and interest.

## Discussion

### A Nuanced Picture

Following the idea that joint music making provides a powerful context for a flourishing life (Lamont and Ranaweera, 2020), this study explored how a community music project, TOSO, that organizes collective free music improvisation sessions with homeless people and individuals with a psychiatric or alcohol/drug background, may contribute to *eudaimonia*. To do so, the PERMA model of well-being was deemed a useful framework as it provides a well-defined set of building block of *eudaimonia*. The results of our study reveal a nuanced picture of the relationship between engaging in this project and the pathway to human flourishing, clearly showing the presence of building blocks of well-being but at the same time revealing the significant presence of negative emotions.

Both interview and video observation data indicate that TOSO creates an environment in which the participants can experience flow, enjoyment, meaningful social relationships and a sense of belonging. We found clear examples of how the project elicits positive emotions, a sense of purpose, engagement and achievement. Such outcomes are described in many similar community music projects (Lee et al., 2017; Schiavio et al., 2019; Vougioukalou et al., 2019; Lamont and Ranaweera, 2020), alongside the emancipatory power of music can play in such programs (Wright, 2006; Veblen, 2007; Elliott and Silverman, 2015). Also, as found in other studies (e.g., Lee et al., 2017; Vougioukalou et al., 2019), direct connections with others in the group (Relationships) and making sense of life (Meaning), are important features of the TOSO project.

Indeed, in the TOSO project, free (or non-idiomatic) improvisation was used for its potential to encompass different levels of playing and its contingency to develop unpredictable social dynamics and interaction (Clarke, 2005; Monk, 2013). For example, the videos show that improvisation tended to start purposefully, even before the rehearsal. More often than not, people began to play as they came in (coaches and participants) thus creating *in the moment* musical activity. This allowed individuals to directly express emotions, which can be linked to the element of positive emotions (P) in the PERMA model. The unscripted interactions that occasionally came out of this spontaneous music making can be linked to the PERMA element of Relationships. Individual and group flow, linked to the element of Engagement in PERMA, is frequently reported in free improvisation performances (Sawyer, 2006; Hytönen-Ng, 2016). Joint music making and collective free improvisation offers a pathway to the PERMA element of Accomplishmen*t*, through participating in concerts or recordings.

Video footage of concerts showed people enjoying themselves, smiling and interacting. In one of the interviews, a participant stated that he really enjoyed “the atmosphere, performing, entertaining people and the interaction with the audience”(P3).

However, despite the positive elements in the interviews and its confirmation in the videos, an interesting finding was the discrepancy in the interviews about social relationships, revealing some negative affect about the social interaction in the group while at the same time reporting strong feelings of group coherence and belonging. The specific context of TOSO may help to explain this discrepancy. Indeed, all participants have a past, such as homelessness, alcohol/drug abuse, psychological and psychiatric problems. Moreover, within the broader community of the city in which TOSO was organized, the group was seen as problematic, dangerous and as outcasts (De Standaard, 2010; *25% more registered cases of homelessness in Ostend*, Adrian, 2012). An important starting point for TOSO was parrying this negative opinion by engaging in a musical practice and as such create conditions which can help people thrive (Wright, 2006, 2019). Possibly, the verbal reflection throughout the interview initiates a form of self-protection or a ‘don’t come any nearer’ mindset. Homeless people with a complex background are often trapped in a cycle which can lead to a maladaptive coping form in terms of alcohol abuse and negative social behavior (Christian and Armitage, 2002; Jarrett, 2010). A study by Christian et al. (2016) revealed the importance of homeless people’s evaluation and beliefs about the benefits of services offered to them. Their findings present an optimistic prospect for interventions to support homeless people. They state: ‘(…) that things that increase social inclusion also increase well-being,’ (p. 25).

However, despite several reports of negative feelings toward other group members, examples of social inclusion were found both in the interviews and observed in the videos. Despite these negative reports, we assume that this form of musical activity can create social bonds and a sense of belonging. It shows that the path to *eudaimonia* is not a linear path. Also, not all community music projects are always a complete success story. In a way, this contradicts the view that all the aspects of well-being (as defined in the PERMA model) should be equally balanced. Indeed, in a recent article Seligman (2018) raises the question of whether an increase in one element of the model can influence other elements of well-being (Seligman, 2018). Also, there seems to be a high correlation between the elements of PERMA. For example, Seligman states that “there are likely causal connections and third variable connections among the elements, e.g., people who find their work meaningful likely accomplish more and people who had a warm childhood likely have better relations and more positive emotions” (Seligman, 2018 p.334). In line with this, we noted an overlap between the elements of the model. Our data show that many participant quotes fitted different elements of the PERMA model. For example, the element of Engagement (flow) encompasses Positive emotion and Accomplishment. This is not surprising, considering dimensions of flow such as a balance between skill and challenge (Accomplishment) and enjoyment (Positive emotion) (Csikszentmihalyi, 1990).

Another interesting finding was the low number of quotes concerning the element of Engagement, especially since the videos clearly display the presence of behavior that indicates such engagement. The element of Engagement which clearly relates to the concept of flow, is considered an important feature of musical activity, especially in improvisation (e.g., O’Neill, 1999; Custodero and Stamou, 2006; Sawyer, 2006; Biasutti, 2015, 2017; Bishop, 2018). Therefore, the low number of engagement quotations was somehow surprising.

However, this may be explained by the interview questions, which did not include specific questions relating to the experience of engagement (or flow), as it is used, for example, in instruments like the flow scales (Jackson and Eklund, 2004) or the PERMA profiler scale (Butler and Kern, 2016). As we stated earlier, the interviews were conducted in an open, spontaneous way to obtain an egalitarian and unforced contact. The low number of quotes does not necessarily imply a lower relevance or even absence of this element. As will be described in the video discussion below, a number of observable flow indicators (Custodero, 2005; Custodero and Stamou, 2006) came to the foreground, such as deliberate gestures, peer awareness, sustained attention and trance.

### Musical and Social Aspects of the Videos

Reviewing the video recordings of performances and rehearsals showed a number of features which are related to the elements of the PERMA model, especially to Engagement (flow) and Relationships.

Biasutti (2015, 2017) stated that it is important to create an environment that might facilitate the occurrence of flow and proposed a number of educational strategies to obtain this. Among them, and relevant for the TOSO project, are the prevention of interruptions during rehearsals in order to keep the concentration. The videos of rehearsals show a quasi non-stop playing, while everybody slowly is drawn into the music. Additionally, creating an environment which allows for musicians to “freely express their creativity” (Biasutti, 2015, p. 9) can lead to group flow. The approach in TOSO, as described above, allowed for this free expression of the participants. The video footage of TOSO showed a number of observable flow indicators (Custodero and Stamou, 2006) and, non-verbal musical and social aspects that were less visible in the interviews. In some cases, the negative participant reports regarding the social and musical outcomes were contradicted positively in the video recordings. We grouped these findings into five themes: corporeal activity, enjoyment and trance, peer awareness, musical interaction, and social contact.

*Joint corporeal activity* was frequently observed during rehearsals and especially during concerts. We noticed embodied engagement and overt bodily expressions. This aligns with recent findings on the embodied nature of musical interaction (Leman, 2016).

*Enjoyment* is seen as a hedonic experience which can be distinguished from related concepts such as happiness or pleasure. According to Lin et al. (2008) enjoyment has three distinct features: to engage in an activity (focused, concentrated), a positive affect (feeling happy, having fun) and fulfilment (useful, rewarding). Before, during and after rehearsals and performances we observed moments of joy and fun related to the positive emotion element of the PERMA model.

A number of sequences showed participants were *absorbed* in the musical activity, sometimes as if they were in a state of *trance*. We see this as an individual experience of flow. They are observations of moments where musicians do not remember what they played, they are *in the moment*, beyond the conscious mind, in the *zone* (Werner, 1996; Hytönen-Ng, 2016). This aligns with Kenny and Gellrich’s (2002) description of improvisation as a transcendental state. In a study, comparing the levels of flow between elite musicians and top athletes, Habe et al. (2019) found a deeper level of transformation of time, which is seen as a typical aspect of music: “Music is developing over time and can induce trance and altered states of consciousness” (p. 5).

*Peer awareness* is an important feature of social interaction and a crucial element in flow (Custodero, 2005; Custodero and Stamou, 2006). In a music setting where improvisation is used, peer awareness leads to a collaborative practice and a shared meaning about the music. Peer awareness ensures that other musicians see, hear and feel musical signals and can respond to them. This can be seen as a form of participatory sense-making (De Jaegher and Di Paolo, 2007) where social interactions are based on interpersonal alignment and coordination (e.g., by imitating each other). Such embodied process occurs partly unconsciously (e.g., through the synchronization of breathing and heartbeat) (Leman, 2016). Being aware of the others while performing plays a role in coordination and collaboration (e.g., building a joint groove, timing, phrasing) and communication (e.g., by giving visual and auditory cues, eye contact) (Davidson and Broughton, 2016). Since it can safely be said that in improvisation there are few certainties of what will come, there is a great responsibility in reacting expressively together. Making eye contact and using all kinds of physical cues are moments that are greatly appreciated by the musicians and the audience. Borgo (2007) rightly notes that an improvising group is more than the sum of its individuals through the dynamic and interactive aspects of the more open improvisational practices and because the process of interaction leads to a joint creative output.

Several authors point out that the *collaborative interaction* in improvisation leads to the generation and evaluation of musical ideas (Aragon and Williams, 2011), group flow (Sawyer, 2006; Biasutti, 2015), musical challenges (Kenny, 2014) and better coordination and tuning (Bishop, 2018). Clarke (2005) argues that non-idiomatic improvisation or free improvisation offers an excellent opportunity to develop unpredictable social dynamics and interaction. We see this interaction during improvisation as a process that is accessible to everyone. It does not require advanced musical and technical qualities. This aspect of improvisation offers an egalitarian view on musical expression based on the exchange and negotiation of new ideas. Although you can perfectly improvise on your own, it is usually something you do together, making it a social interaction in which the participants have the same goal: to build a musical whole together.

Thus, improvisation is about creating a common meaning through the unfolding (emergence) of the interaction. In other words, it can be a pathway to *eudaimonia*.

*Social connection* to others was observed mainly before and after rehearsals and performances. This refers to the Relationship element of the PERMA well-being model (Seligman, 2011), which is about the creation of positive connections with others.

Positive and respectful engagement leads to the formation of bonds and friendship. Such behavior, the capacity of connecting to others through verbal and non-verbal behavior can create a sense of agency and lead to well-being. This was also reported in studies with young children (Webster-Stratton and Reid, 2004).

### The Role of the Coach(es)

In projects such as TOSO, the coaches or facilitators play an important role. The pedagogical-artistic strategies (Hickey, 2015; Schiavio et al., 2019), their training and their beliefs may play a crucial role in facilitating a path to *eudaimonia*. Schiavio et al. (2019) found three aspects regarding the facilitators in a community music program (Meet4Music) for people with different social and cultural backgrounds: *collaboration* (through egalitarian interaction between facilitators and participants and playing together), the use of *non-verbal communication* (through an embodied use of gestures and facial expressions) and a *sense of togetherness* (being able to connect to the group). These findings coincide with the preliminary finding of our analysis of the strategies used by the TOSO coaches (Verneert et al., 2021). But, as stated in the study of Schiavio et al. (2019), it is important to also include the perspective of the participants, which we explored in this case study.

In the study of Vougioukalou et al. (2019) is stated that a balance is needed between the free improvisation and leadership and structure, in order to enable a group performance.

The absence of structure in TOSO sometimes led to frustration and chaos, as clearly appears, for example, in this quote: “I think that’s such a pity. [thinking] for example, at one concert we agreed to do a minute’s intro with a certain number of instruments. The others don’t play during that minute and then, after that minute, other instruments are added. But after five seconds or so everybody was already playing and that wasn’t really the agreement. Sometimes we all play together without listening to each other. At other times, everything gets ruined because everyone wants to do their own thing. We should listen more to each other, I think”(P6). Nevertheless, this lack of structure was an important feature of the TOSO project, where the process and the performance were interchangeable and unpredictable. It required a radical re-thinking of musical parameters from the TOSO coaches in accepting the musical chaos.

### Collaborative Creativity and Free Improvisation

Using free improvisation allows personal musical expression regardless of the level of technical and musical skills. It provides opportunities for an equal exchange of musical ideas and a way to express emotion. As one participant said: “I wanted to play along and found it really cool, because it is music without limits.” The perceived freedom is an important feature in the project: “Well, yes, you can do your own thing [speaks more softly], without those endless comments, telling you do it like this or that. So, you can actually express yourself and experience that feeling of freedom.”

At an individual level, the use of free musical improvisation acted as a relief from stressors, ‘letting go,’ do an emotional outlet. This becomes clear for instance in the following quote from one of the participants: ”That creative outlet, being able to perform. It’s different, it’s free jazz, you improvise as much as you can. In a choir you are stuck to scores, lyrics and all. In TOSO you experience the music differently.”

It is clear from research and theory that free musical improvisation offers a space for *collaborative creativity* (Borgo, 2007; Kenny, 2014; Hickey, 2015). Engaging in this activity provides opportunities to connect to others and form social bonds through music making. Wilson and MacDonald (2019) state that studying community music programs for disadvantaged people must consider social and psychological features, next to musical features. The joy of participating in a community music program and the social relationships that are formed, is seen as a perquisite for collaborative creativity (Kenny, 2014; Wilson and MacDonald, 2019).

As found in other studies (e.g., Lee et al., 2017), the element of Relationships is the most cited. The process of creating meaning through the dynamic and interactive aspects of free improvisation led to strong social outcomes and bonding, as exemplified in the following statements: “The music cements friendships. There are different cultures. You may not understand the language, yet you understand them through the music”(P12). Or: “It means a lot. (silence) a lot, a lot to me. Not just the friendship. It’s like coming home“(P8).

The importance of the group was clearly stated in the quotes, but also reflected in the video footage. Performing music in a group can lead to the experience of flow and “enhances the transformation of time” (Habe et al., 2019 p. 6).

Recently, Lamont and Ranaweera (2020) found anxiety and negative emotions toward performance in a group of amateur musicians. The positive emotional and social outcomes seemed to counter these negative feelings. Based on the interview and video observation data, we did not observe any differences between performances and rehearsals. Feelings of anxiety toward performing were not reported. This might be because of the particular approach of TOSO, where no difference is made between *the street and the stage*.

More research imposes itself to explore this further, in an effort to see if this is related to the specific features of the group or the result of individual personality traits. Also, because some research states that working toward a music performance or an end product is of greater value and importance to the participants than the musical process itself (Hallam and Creech, 2016; Lamont and Ranaweera, 2020).

### Reflections on the Methodology

In this project, we used interviews and video observations because we felt they would complement each other in an effort to capture the complexity of community music projects with homeless people (Aragon and Williams, 2011; Gebauer, 2011). In this particular instance, both methods proved challenging because of the specific problems most participants were facing. In other words: putting them in front of a camera and recording rehearsals/performances or doing a structured interview was not feasible and would even possibly interfere with the validity of the recordings and interviews. That is why we opted for more informal video recordings and a documentary style as valuable information gatherers to gain deeper insights into how participants experience TOSO. And even, then, sometimes the use of the camera elicited negative reactions. Evidently, we acknowledge the limitations of such recordings, as they are idiosyncratic and not embedded within a systematic video footage. Indeed, a recording of every rehearsal would allow to observe the process in a more continuous way, thereby providing a more encompassing view on the building blocks of *eudaimonia*. However, we are convinced that the ‘witnessing’-approach is an interesting path to follow and that ways of gathering information from the inside of the experience should be developed, rather than taking on an outsider perspective through systematic video recording. Working with occasional and spontaneous recordings, like participants recording with a mobile phone during rehearsals, could provide a *first-person perspective*, where the camera becomes part of the activity. This makes it possible to grasp the embodied knowledge of the participants, feel the dynamics of the interaction and sometimes, underlying resistance. Thus capturing the project from inside, may generate new ideas. It also counterbalances the commodification of music as a mere tool to work with disadvantaged groups. Moreover, spontaneous recordings allow to go beyond the boundaries in space (e.g., in and outside the rehearsal room) and time (before, during and after the rehearsal) and, as such, to capture the participants experience and behavior in a more extended way.

Making video recordings wasn’t straightforward, but neither was interviewing the participants. It required an informal stance in an effort not to make participants feel they were being ‘researched.’ While this limited systematically addressing specific topics, we believe the informal approach is valuable. In our view, the fact that participants felt comfortable expressing negative aspects, illustrates this.

Our future work will further address these specific challenges. In our view, research with people in adverse situations requires an innovation of research methods.

Research into community music programs reports most frequently on positive outcomes of such programs, illustrating how the experience of playing music together and improvising has great potential to improve well-being. However, our study provides a more nuanced picture, showing that negative feelings toward the project and the group can actually coexist with feelings of enjoyment. The positive feelings may well be more or less evoked in the moment of playing, while diminished when the music stops. Even so, the experience of collaborative creativity and being part of the group while making music is a powerful step to empowerment, self-actualization and *eudaimonia*.

Finally, the primary researcher (first author) participated as a musician in some of TOSO’s rehearsals and performances. This established a ‘down to earth,’ equal relationship with the participants, who did not see him as an external researcher but as one of the musicians. We feel this approach also deserves to be further explored and developed. Like the informal approach in the interviews and recordings, the researcher’s insider perspective may help establish a veridical image of the nature of such projects. We understand that this may lead to a dynamic tension between participating and researching. However, it does make it possible to explore the boundaries of the ways such projects are investigated, just as the TOSO project explores the ways in which music and improvisation is usually conceived. This could lead to interesting methodological developments in which the insider and outsider perspective could be merged, and artistic and empirical research join forces.

## Conclusion

According to different scholars (Lee et al., 2017; Schiavio et al., 2019; Lamont and Ranaweera, 2020), there is a need for in-depth case studies to address the topic of music programs and well-being.

In this study, systematic qualitative analysis and subjective interpretations of occasional video recordings of a community music project were combined in an effort to elaborate on the relationship between the concept of *eudaimonia*, as defined by Seligman (2011) and the interactive dimensions of collective group improvisations.

An interesting finding was the discrepancy in the interviews as to social relationships, revealing some negative affect about the social interaction in the group while at the same time reporting strong feelings of group coherence and belonging. The results, thus, reveal a nuanced picture of the relationship between engaging in a community music project that revolves around homeless people and individuals with a psychiatric or alcohol/drug related background and the pathway to human flourishing (or *eudaimonia*).

This research shows how musical encounters can contribute to *eudaimonia*, motivating people with diverse backgrounds to take a life-long positive engagement in music and have them experiencing a ‘sense of belonging’ throughout their joint musical development.

However, our study also points out the complexity of this type of context and the methodological challenges it poses. For example, the discrepancy between some interview content and observations in the videos highlighted the need to combine interviews and video observation, but also that the participants’ specific situation calls for adapted methods. We believe this is an important part of our future work - namely, to further develop the interview method and the way video recordings are used as a form of ‘witnessing.’

## Data Availability Statement

The raw data supporting the conclusions of this article will not be made publicly available because the terms of consent for research participation did not specify that data would be accessible other than by the research team. The initial coding is accessible at LUCA School of Arts, Leuven, Belgium. Requests to access this dataset should be directed to filip.verneert@luca-arts.be.

## Ethics Statement

Ethical review and approval was not required for the study on human participants in accordance with the local legislation and institutional requirements. The patients/participants provided their written informed consent to participate in this study.

## Author Contributions

FV and LN made the equal contribution. TD contributed as last author. All authors contributed to the article and approved the submitted version.

## Conflict of Interest

The authors declare that the research was conducted in the absence of any commercial or financial relationships that could be construed as a potential conflict of interest.

## Appendix 1

**Questions Semi-Structured Interviews Participants TOSO**

Can you tell something about yourself?

How did you end up in TOSO?

What do you like about TOSO?

Has participating in TOSO group an impact on the social contacts between the participants?

Do you meet with or talk to others of the group outside of TOSO moments?

Is making music within a group important to you?

Are there conflicts in the TOSO group?

Is there a difference between the members who have been coming to rehearsals for years and these who have only just joined TOSO?

What is the approach of the coaches during rehearsals?

What does TOSO mean to you?
